# Impact of time in therapeutic range (TTR) within the first 72 h on prognosis in patients with pulmonary embolism treated with unfractionated heparin

**DOI:** 10.1007/s11239-025-03167-2

**Published:** 2025-08-14

**Authors:** Amit Ifergan, Ranel Loutati, Ariella Tvito, Mony Shuvy, Shemy Carasso, Dana Deeb, Louay Taha, Mohammad Karmi, Mohammed Manassra, Akiva Brin, Ofir Rabi, Noam Fink, Pierre Sabouret, Amro Moatz, Abed Qadan, Nir Levi, Tali Bdolah-Abram, Michael Glikson, Elad Asher

**Affiliations:** 1 Jesselson Heart Center, Shaare Zedek Medical Center, The Eisenberg R&D Authority, and Faculty of Medicine, Hebrew University of Jerusalem, Jerusalem, Israel; 2 Assuta Medical Centers, Faculty of Medicine, Hebrew University of Jerusalem, 6329302 Tel Aviv, Jerusalem, Israel; 3 ACTION Study Group, Institut de Cardiologie, Hôspital Pitié-Salpêtrière, Sorbonne Université, 75005 Paris, France; 4 National College of French Cardiologists, 13 Rue Niepce, 75014 Paris, France; 5 Faculty of Medicine, Hebrew University of Jerusalem, Jerusalem, Israel

**Keywords:** Pulmonary embolism, Heparin, Time in therapeutic range, Acute cardiovascular care

## Abstract

Pulmonary embolism (PE) is a life-threatening condition often treated with unfractionated heparin (UFH) in intermediate high-risk patients. Activated Partial Thromboplastin Time (aPTT) is used to monitor UFH efficacy. We sought to evaluate the correlation between time in therapeutic range (TTR) and prognosis in patients with pulmonary embolism treated with unfractionated heparin. A prospective cohort study included 203 patients admitted to a tertiary care center between July 2019 and August 2024 with a confirmed diagnosis of intermediate risk PE treated with UFH. TTR was calculated based on aPTT values during the first 72 h of hospitalization. The correlation between TTR and mortality rates was assessed. Out of the 203 patients, 116 (57%) achieved therapeutic range at least once, with a mean TTR of 43.1% (± 22.4) and a median of 39%. Nevertheless, the overall mean TTR for all patients was 24.6% (± 27.3), with a median of 18.8%. During the study period 25 (12.3%) patients have died, of them 9 (4.4%) within 30 days and 16 (7.9%) within one year. Higher TTR was associated with reduced 30-day (p = 0.051) and one-year (p = 0.045) mortality rates. Receiver Operating Characteristic (ROC) analysis identified a TTR threshold of 21.5% for predicting one-year mortality, demonstrating a high negative predictive value (NPV) of 96.8% but a low positive predictive value (PPV) of 12%. Patients with acute PE who achieved higher TTR exhibited better outcomes at 30 days and one year. However, most patients did not reach adequate TTR levels, leaving its role as an independent prognostic indicator uncertain. Larger studies are necessary to optimize therapeutic strategies and improve outcomes in intermediate-risk PE patients.

**Supplementary Information:**

The online version contains supplementary material available at 10.1007/s11239-025-03167-2.

## Introduction

Pulmonary embolism (PE) is a critical condition characterized by the sudden obstruction of a pulmonary artery by a blood clot typically originating from deep veins in the legs. This obstruction severely compromises blood flow to the lungs, leading to potentially life-threatening consequences. [1–3].

Management of acute PE focuses on stabilizing the patient, preventing further clot development, and minimizing the risk of recurrent embolism [2]. Immediate treatment involves the administration of anticoagulant medications, such as unfractionated heparin (UFH), low molecular weight heparin (LMWH), fundaparinux and direct oral anti-coagulants (DOACs), to prevent clot growth[1, 2]. [4] In intensive cardiovascular care units (ICCU), UFH is frequently recommended according to guidelines, especially in cases of hemodynamic instability, intermediate/high-risk PE where primary reperfusion may be required, renal impairment (creatinine clearance ≤ 30 mL/min), or obesity.

Activated Partial Thromboplastin Time (aPTT) is used to monitor UFH therapy by measuring clot formation time in the intrinsic and common coagulation pathways. [5] Subtherapeutic aPTT is defined as a value less than 60 s whereas high aPTT is defined as greater than 85 s. [6] Low or high aPTT can cause further clot formation or bleeding respectively [5].

Although balancing aPTT levels can be challenging, data regarding therapeutic aPTT with UFH to treat patients with PE is scarce. One recent study found the proportions of patients in the therapeutic range was 26.3–33.1% in the first 24 h for patients treated with a bolus and infusion of UFH, and 31.6–51.9% for the first 24 h for titrated infusion only [6]. Another study found that 60% of patients failed to reach therapeutic aPTT within the first 24 h of UFH therapy, with a 30-day mortality rate of 10.7% (5.6% for patients with therapeutic aPTT vs. 14.8% for those with subtherapeutic levels) [7]. Despite these findings, data regarding aPTT and prognosis in PE patients is scares, moreover, most of the data is limited to either a single measurement of aPTT or to the first measurement within the therapeutic range [6, 8, 9]. There is currently very little information regarding time in therapeutic range (TTR), which represents the coagulation balance over time in a more continuous way, and its relation to prognosis of PE patients. Hence, we sought to evaluate the correlation between the TTR for UFH treatment and prognosis in acute PE patients admitted to a tertiary care center ICCU.

## Methods

### Study population

All patients diagnosed with acute intermediate-high risk PE who were admitted to a tertiary care ICCU at the Shaare Zedek Medical Center between July 2019 and August 2024 and were treated with UFH for acute PE were prospectively enrolled. The diagnosis of PE was based on symptoms and computed tomographic pulmonary angiography (CTPA) in accordance with contemporary ESC guidelines [1]. Intermediate–high-risk pulmonary embolism (PE) patients are those who are haemodynamically stable at presentation (i.e., no shock or sustained hypotension) but show evidence of both right ventricular (RV) dysfunction and myocardial injury [1]. Patients were excluded if they had alternative diagnosis such as pericarditis or acute coronary syndrome, if they were low-risk patients treated with LMWH, or if they were high-risk patients who underwent thrombolytic therapy.

### Data collection

Data was anonymously documented in the ICCU by the local coordinator and prospectively submitted into an electronic case report form. Data were checked for accuracy and out‐of‐range values by the coordinating unit. Demographic information, presenting symptoms, comorbid conditions, and physical examination were systematically recorded. Additional data included laboratory results, imaging studies, angiographic findings, and details of the clinical course. Each patient was followed-up for 12 months.

### aPTT measurement and time in therapeutic range

Each patient received an initial bolus of UFH (4000–5000 IU) followed by a continuous titrated infusion starting at 18 units/kg/hour. Heparin dosing was managed using a standardized protocol embedded in the electronic medical record (Table1).

### aPTT assey was made by using SynthASil-0020006800 by HemosIL

TTR was calculated as the percentage of time a patient’s aPTT values remained within the therapeutic (60-85 s). For this calculation, it was assumed that changes between two consecutive aPTT measurements occurred linearly, hence, the TTR was calculated throughout each patient’s test during hospitalization.

For each patient during hospitalization, 1–17 aPTT tests were taken in 72 h. TTR is calculated as the percentage of total monitored time during which a patient’s aPTT values are within the defined therapeutic range. This is typically determined by linear interpolation between consecutive aPTT measurements, assigning each time interval to the corresponding aPTT value, and summing the time spent within the therapeutic range over the total observation period (72 h) [10].

Time above the therapeutic range was defined as the percentage of time a patient’s aPTT values exceeded 85 s, while time below the therapeutic range was defined as the percentage of time aPTT values fell below 60 s.

Each patient’s TTR was constructed from the set of aPTT measurements taken during the first 72 h following admission to the ICCU.

### Ethics and consent

This study complied with the Declaration of Helsinki and has been approved by the Shaare Zedek Medical Center Institutional Review Board (IRB) (IRB protocol number SZMC-0270–23). Consent was deemed unnecessary in accordance with national regulations. The study received no funding, and the authors have no conflicts of interest to declare. Additionally, all methods were conducted in compliance with the relevant guidelines and regulations.

### Statistical analysis

Quantitative variables are presented as mean ± Standard Deviation (SD), as well as median and interquartile range (25–75%). Categorical variables are presented as frequencies and percents. In order to test the association between two categorical variables, the χ [2] test as well as the Fisher’s exact test were used. The comparison of a quantitative variable between two independent groups was performed by using the non-parametric Mann–Whitney test (M-W). The Pearson correlation coefficient and the Spearman non-parametric correlation coefficient were calculated for estimating the strength of the association between two quantitative variables. The non-parametric tests were applied for variables which were not normally distributed.

The multivariate logistic regression model was applied for simultaneously assessing the effect of several independent variables on a dichotomous dependent variable. This model was applied using both the"enter"method and the forward, stepwise, likelihood ratio method. All odds ratio (OR) presented are adjusted. ROC analysis was applied in order to find the best cutoff point of a quantitative variable, for distinguishing between mortality/no mortality. The cutoff point was based on the Youden’s index which finds the optimal combination of sensitivity and specificity, taking them into account equally. Based on the cutoff point, the sensitivity, specificity, PPV and NPV of the test were calculated.

All statistical tests were two-tailed, and a p-value of 5% or less was considered statistically significant.

Statistical calculations were performed using IBM, SPSS Statistics, version 29.

## Results

### Patient characteristics

During the study period, a total of 203 consecutive patients were enrolled. The mean age was 65 ± 17.8 years, and 114 patients (56.2%) were female. Among the participants, 77 (38%) had hypertension, 75 (37%) had dyslipidemia, 44 (22%) had diabetes mellitus, 21 (10%) had chronic obstructive pulmonary disease (COPD), and 44 (22%) were active smokers. Detailed patient characteristics are summarized in Table 1.

**Table 1:** Patients’ characteristics

	All patients	Achieved TTR	Didn’t achieve TTR
N	203	116	87
Age (mean, median)	65 ± 17.8, 69 (53–78)	66.3 ± 16.4, 69.5 (55.2–76.7)	63.2 ± 19.4, 67 (49–78)
Sex (female)	114 (56.2%)	60 (51.7%)	54 (62.1%)
BMI (mean, median)	30.14 ± 6.9, 28.7 (25.7–33.1)	30.5 ± 7.1, 28.7 (25.9–33.1)	29.7 ± 6.5, 28.7 (24.3–33.2)
Hypertension	77 (38%)	42 (36.2%)	35 (40.2%)
Dyslipidemia	75 (37%)	47 (40.5%)	28 (32.2%)
Diabetes mellitus	44 (21.7%)	29 (25%)	15 (17.2%)
Asthma/COPD	21 (10.3%)	13 (11.2%)	8 (9.2%)
Active/past malignancy	38 (18.7%)	17 (14.7%)	21 (24.1%)
Smoking	17 (8.4%)	9 (7.8%)	8 (9.2%)
CVA/TIA	15 (7.38%)	9 (7.8%)	6 (6.9%)
Prior CAD	16 (7.88%)	12 (10.3%)	4 (4.6%)
Past PE	13 (6.4%)	4 (3.4%)	9 (10.3%)

BMI, body mass index; COPD, chronic obstructive pulmonary disease; CVA, cerebro vascular accident; TIA, transient ischemic attack; CAD, coronary artery disease; PE, pulmonary embolism

## Time in therapeutic range

One hundred and sixteen (57%) patients were in therapeutic range at least once with a mean TTR of 43% and a median TTR of 39% (24.6–60.2%). Nevertheless, the mean TTR of all patients was only 24.6 ± 27.3% with a median TTR of 18.8% (0–45.3%).

### Time above and below therapeutic range

One hundred and two patients (50%) were above the therapeutic range at least once. The mean time above therapeutic range is 18.5% ± 24.4%, median 1.1% (0–33.7%). In contrast, 179 patients (88%) were below the therapeutic range at least once, The mean time below therapeutic range 56.9% ± 36.3%, median 57.3 (25.6–100%).

### Time in therapeutic range and other variables

No statistically significant correlation was found between TTR and age, body mass index (BMI), albumin, high-density lipoprotein (HDL), low density lipoprotein (LDL), C reactive protein (CRP), hemoglobin A1C, thyroid stimulating hormone (TSH), white blood cells (WBC) count, hemoglobin (HB) level, platelets (PLT) count, lactate, international normalized ratio INR, fibrinogen, troponin and D-dimer.

The mean and median TTR values were higher in men compared to women: 31.8% ± 30.9% and 30.1% (0–60.4%) vs. 19% ± 22.7% and 9.19% (0–37.9%) (p < 0.01 for both). However, the mean and median time above the therapeutic range were higher in women than in men: 22.5% ± 27.2% and 11.7% (0–39.9%) vs. 13.4% ± 19.1% and 0% (0–24%) (p < 0.05 for both).

## Mortality

During the study period 25 (12.3%) patients have died, of them 9 (4.4%) within 30 days and 16 (7.9%) during the first year, 11 patients died after the first year.

**Fig. 1:**
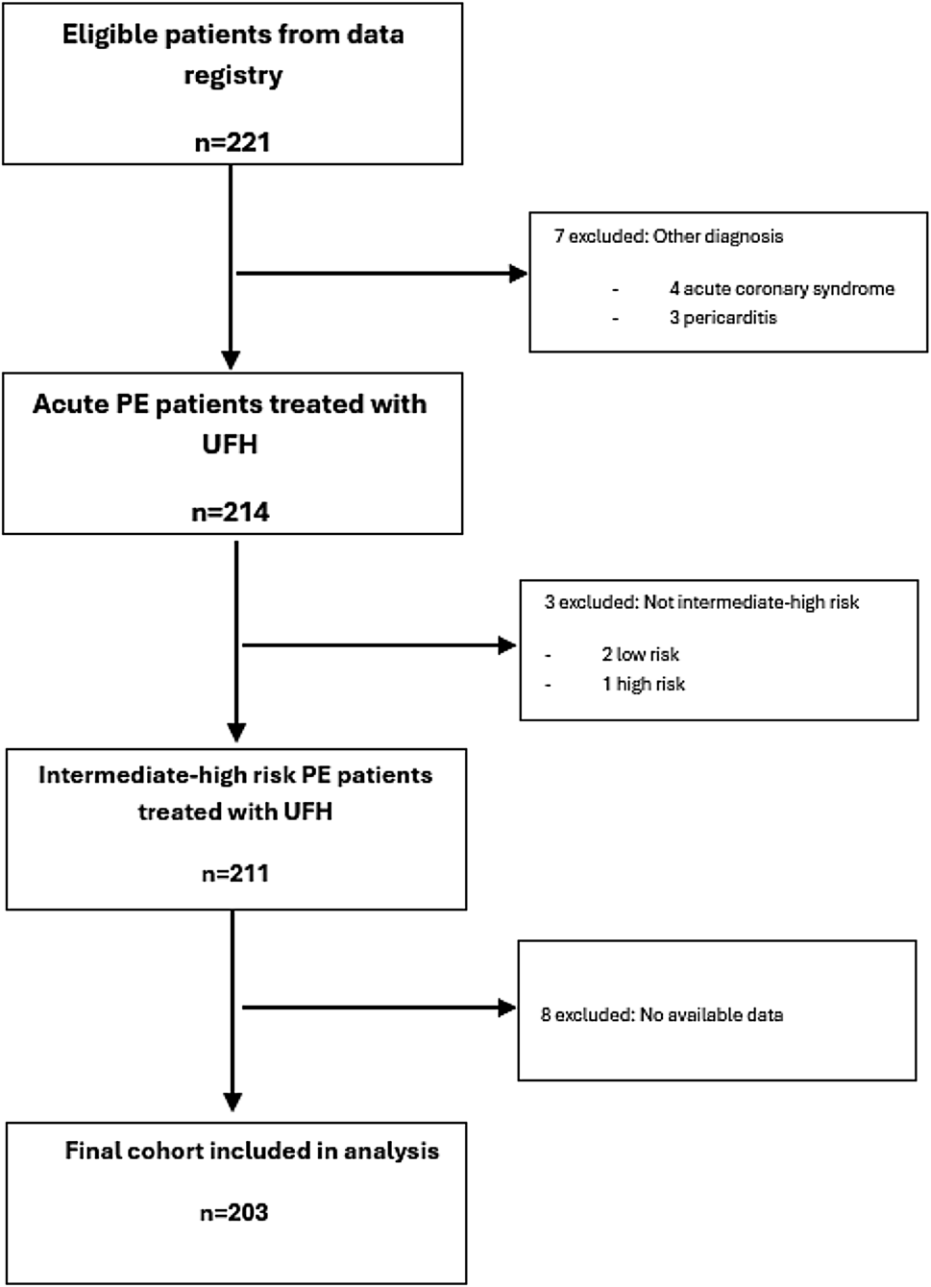
Study cohort selection flowchart

The mean and median TTR was significantly lower in patients who died within the first 30 days compared to those who survived (mean—9.5% ± 22.1% vs. 25.3% ± 27.4%, p = 0.05, median – 0% (0–10.1%) vs 19% (0–45.6%), p < 0.05) Similarly, the mean and median TTR was lower in patients who died within the first year compared to survivors (mean—12.9% ± 22.6% vs. 25.6% ± 27.5%, p = 0.045, median – 0% (0–21.1%) vs. 19% (0–45.8%), p < 0.05) (Fig. 1). The one-year mortality rate was higher in women than in men (11.4% vs. 3.4%, p = 0.035).

### Multivariate analysis for 1 Year Mortality

In a multivariate logistic regression analysis, the significant variables predicting mortality were age (*p* = 0.031, OR = 1.059, CI 1.005–1.116), albumin level (*p* = 0.003, OR = 0.1, CI 0.026–0.458), and the presence of malignancy (*p* = 0.011, OR = 4.911, CI 1.444–16.704). However, time in therapeutic range (TTR) was not found to be a significant predictor of mortality (*p* = 0.337, OR = 0.986, CI 0.959–1.014).

### ROC curve analysis of TTR value and 1 year mortality

**Fig. 2:**
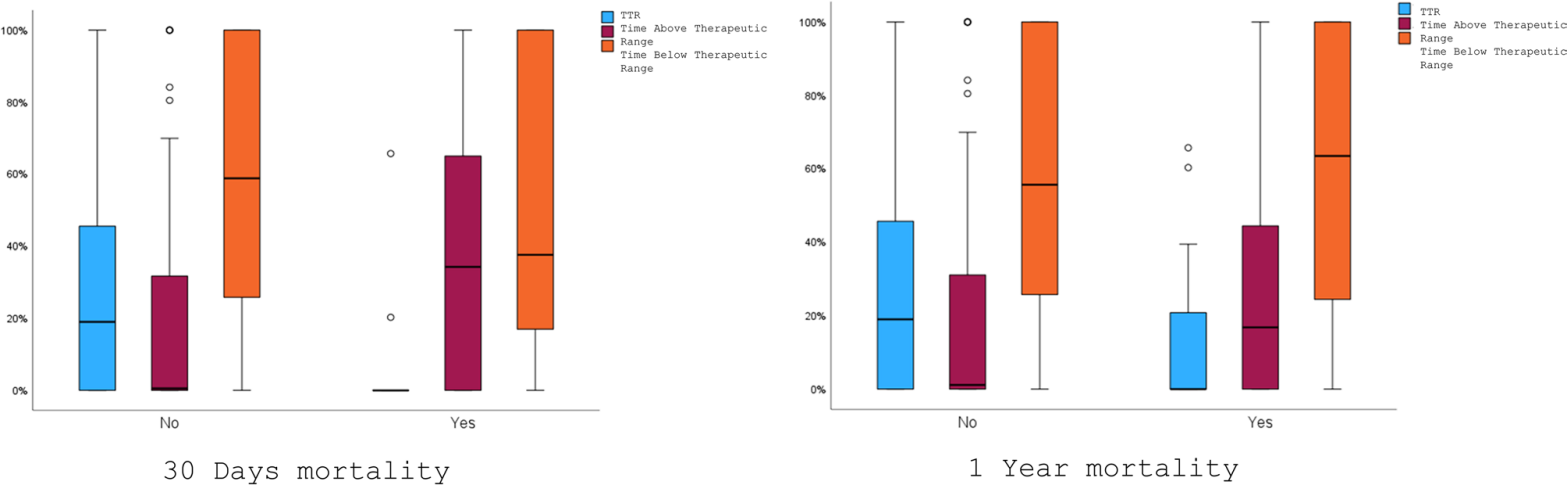
30 Days & 1 Year mortality and TTR

ROC curve analysis identified an optimal cutoff TTR at 21.5%. The area under the ROC curve was 0.645, with a sensitivity of 81.3%, specificity of 49.2%, a poor positive predictive value (PPV) of 12%, and an excellent negative predictive value (NPV) of 96.8% (Fig. 2). When conducting a multivariate analysis of one-year mortality with TTR as a categorical variable, based on the cutoff from the ROC curve, the p-value was 0.084 (Table 2).

**Table 2:** Multivariate Analysis of 1 Year Mortality with TTR as a Categorical Variable

	Significance	Adjusted OR	95% C.I. for OR	
			Lower	Upper
Age	0.33	1.057	1.005	1.113
Albumin	0.002	0.099	0.022	0.433
Malignancy	0.014	4.723	1.361	16.391
TTR, cutoff 21.5%	0.084	3.417	0.849	13.757

OR odds ratio, C.I confidence interval, TTR time in therapeutic range

## Discussion

Our study explores the relationship between TTR during UFH therapy and prognosis in patients with acute PE admitted to a tertiary care center ICCU. Given the clinical challenge of balancing therapeutic aPTT levels, the findings contribute valuable insights into optimizing anticoagulation management in acute high-risk PE cases. Our main findings are: a) Therapeutic Range Achievement: Only 57% of patients achieved therapeutic aPTT at least once during their hospital stay, with a mean TTR of 24.6%. b) Gender differences in TTR: Men demonstrated higher mean and median TTR values than women. Conversely, women spent more time above the therapeutic range. c) Mortality and TTR Correlation: Lower TTR was significantly associated with increased 30-day and 1-year mortality rates. The mean TTR for patients who died within the first 30 days (9.5%) and 1 year (12.9%) was substantially lower than survivors. However, TTR did not emerge as an independent predictor of mortality in multivariate analysis, suggesting its role may be secondary to other factors, such as age, albumin levels, and malignancy.

### Therapeutic range achievement

Only 57% of patients achieved therapeutic aPTT levels at least once during hospitalization, with a mean TTR for this group of 43%. Nevertheless, total patients mean TTR was only 24.6%. The low overall TTR suggests a substantial gap in maintaining effective anticoagulation within the therapeutic window which is supported by previous studies suggests that a significant proportion of patients fail to achieve therapeutic aPTT within the critical 48 h, with only 26.3–33.1% of patients reaching the range during this period [6]. Similarly, another research reported that 60% of patients did not achieve therapeutic aPTT level within 24 h. [7].

These findings highlight the challenge of UFH titration in critical care settings, especially for acute PE patients with complex profiles such as renal impairment or obesity. [1].

### Gender differences in TTR

**Fig. 3:**
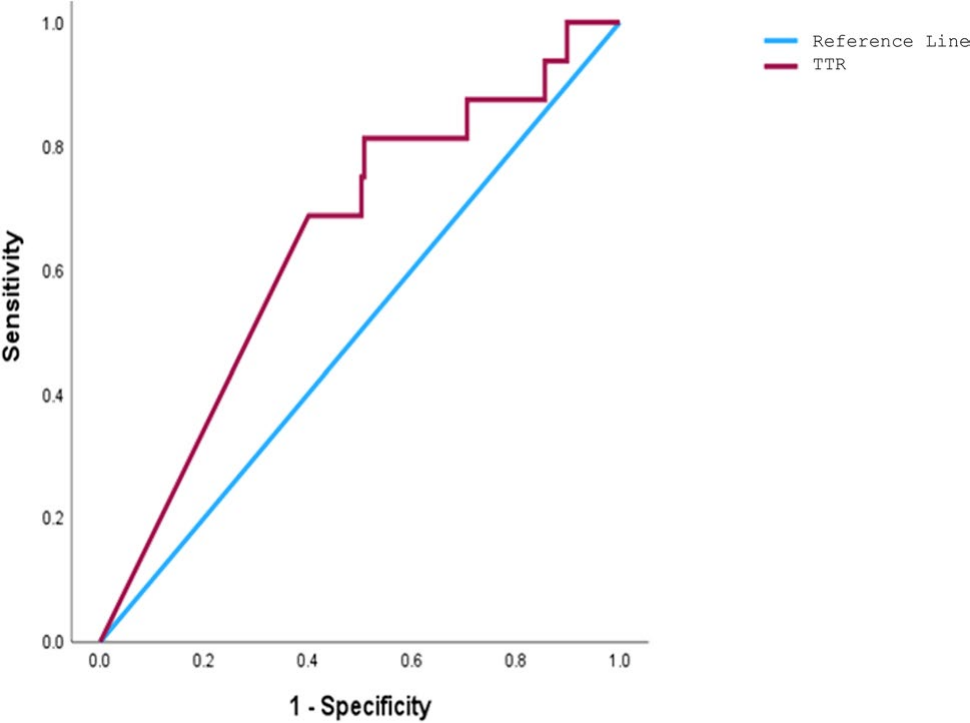
ROC Curve analysis of TTR value and TTR

Men demonstrated higher mean TTR compared to women (31.8 ± 30.9% vs. 19 ± 22.7%) while women spent more time above therapeutic range (22.5 ± 27.2% vs. 13.4 ± 19.1%). This is in line with previous studies that found that women had higher heparin levels and aPTT values than men after receiving the same heparin doses, indicating a heightened sensitivity to heparin in females [11]. Similarly, research on heparin administration during carotid endarterectomy reported that females reached significantly higher levels of anticoagulation compared to males [12]. These differences could stem from physiological variations or disparities in anticoagulation response, warranting further research to tailor UFH dosing more effectively based on sex-specific factors (Fig. 3).

### Mortality and TTR correlation

Lower TTR was significantly associated with increased 30-day and 1-year mortality rates. The mean TTR for patients who died within the first 30 days (9.5%) and 1 year (12.9%) was substantially lower than survivors. However, TTR did not emerge as a strong independent predictor of mortality in multivariate analysis, suggesting its role may be secondary to other factors, such as age, albumin levels, and malignancy. ROC curve analysis identified a TTR cutoff of 21.5% with high sensitivity (81.3%) and high negative predicting value (96.8%) but poor positive predictive value (12%) for 1-year mortality. TTR as a screening tool remains questionable because it does not consistently identify patients at high risk of death (low PPV).

Previous studies found that patients who achieved therapeutic aPTT within 24 h had improved mortality and fewer bleeding events, emphasizing the importance of timely therapeutic anticoagulation [9]. Nevertheless, this might be due to the fact that patients who achieved therapeutic aPTT were less complicated than patients who did not achieve sufficient therapeutic aPTT and had more comorbidities and more complicated PE. More complicated patients can find difficulties in achieving therapeutic aPTT due to heparin resistance that can be caused by antithrombin III (ATIII) deficiency or elevated levels of factor VIII and fibrinogen, which are common in critically ill patients [13]. Additionally, comorbid conditions can significantly affect anticoagulation therapy. For example, liver disease can alter the metabolism of anticoagulants, and conditions like vitamin K deficiency can impact coagulation pathways, making it more challenging to maintain therapeutic aPTT levels. [14].

This highlights TTR’s potential utility as a screening tool to identify high-risk patients while underscoring its limitations as a standalone predictor. Moreover, the multivariate mortality predictors such as age, albumin levels, and malignancy were significant independent predictors of 1-year mortality. These findings reinforce the multifactorial nature of outcomes in PE patients and the need for holistic risk stratification beyond TTR alone. [15].

### Comprehensive assessment of TTR

Unlike earlier studies that focused on single or initial aPTT measurements, our study evaluates TTR over the first 72 h of hospitalization. By using a continuous assessment of aPTT levels, the study offers a more dynamic understanding of anticoagulation balance and its impact on patient outcomes. Moreover, previous research emphasized the percentage of patients achieving therapeutic aPTT but lacked detailed exploration of TTR’s correlation with mortality. This study bridges that gap, demonstrating significant differences in mean TTR between survivors and non-survivors at 30 days and 1 year. Finally, with a mean follow-up period of 35 months, the study extends its observations beyond short-term outcomes, providing valuable insights into the long-term prognosis of PE patients.

### Clinical implications

This study underscores the need for improved strategies to maintain therapeutic aPTT levels during UFH therapy. Potential approaches include: Enhanced monitoring protocols, such as more frequent aPTT assessments; Development of algorithms to personalize UFH dosing based on demographic and clinical factors; Consideration of alternative anticoagulants (e.g., LMWH or direct oral anticoagulant—DOACs) in eligible patients to overcome the challenges associated with UFH.

### Limitations of aPTT

The concept of an aPTT"therapeutic range"for unfractionated heparin (UFH) is historical and was not validated specifically for pulmonary embolism (PE). The commonly used range of 1.5–2.5 times control was derived from early studies but has since been shown to be unreliable, with significant variation based on reagents and lab methods [16, 17]. aPTT is also influenced by biological and analytical variability—such as acute-phase reactants, liver dysfunction, and inter-laboratory differences—reducing its correlation with heparin activity and clinical outcomes [18–20]. These limitations undermine the utility of aPTT-based TTR as a prognostic tool in PE. Anti-factor Xa monitoring, though not without limitations, is considered a more direct and consistent measure of heparin effect [19, 20]. Given the lack of standardization and weak correlation with outcomes, caution is needed when interpreting aPTT-based TTR in this context.

### Study limitations

Our study has several limitations, First, it was a single center study, which limits generalizability to other populations or settings. Second, the sample size was relatively small for mortality analysis with reduced power to detect subtle associations. Third, we did not include patients who initially received thrombolysis due to shock and had worse outcomes. Finally, Various physiological and pathological conditions, including liver dysfunction, disseminated intravascular coagulation (DIC), factor deficiencies, inflammation, and acute-phase reactants, affect aPTT. These confounders may impact TTR calculations and should be acknowledged when interpreting the results. These factors introduce variability that is independent of heparin dosing, potentially limiting the reliability of TTR as a surrogate marker for anticoagulation quality.

## Conclusion

Although TTR correlates with short- and long-term mortality in PE patients, its role as an independent prognostic marker remains uncertain. Future studies with larger cohorts and broader anticoagulant comparisons are essential to refine therapeutic strategies and improve outcomes for intermediate-high risk PE patients.

## Supplementary Information

Below is the link to the electronic supplementary material.

Supplementary file1 (DOCX 17 kb)

## Data Availability

No datasets were generated or analysed during the current study.

## References

[1] Konstantinides SV, Meyer G, Becattini C, Bueno H, Geersing GJ, Harjola VP, Huisman MV, Humbert M, Jennings CS, Jiménez D, Kucher N, Lang IM, Lankeit M, Lorusso R, Mazzolai L, Meneveau N, Ní Áinle F, Prandoni P, Pruszczyk P, Righini M, Torbicki A, Van Belle E, Zamorano JL, ESC Scientific Document Group (2020) 2019 ESC guidelines for the diagnosis and management of acute pulmonary embolism developed in collaboration with the European respiratory society (ERS). Eur Heart J 41(4):543–603. 10.1093/eurheartj/ehz405 31504429 10.1093/eurheartj/ehz405

[2] Soloff LA, Rodman T (1967) Acute pulmonary embolism. II. clinical. Am Heart J 74(6):829–847. 10.1016/0002-8703(67)90102-0 6073360 10.1016/0002-8703(67)90102-0

[3] Carson JL, Kelley MA, Duff A, Weg JG, Fulkerson WJ, Palevsky HI, Schwartz JS, Thompson BT, Popovich J Jr, Hobbins TE (1992) The clinical course of pulmonary embolism. N Engl J Med 326(19):1240–1245. 10.1056/NEJM199205073261902 1560799 10.1056/NEJM199205073261902

[4] St Pierre BP, Edwin SB (2019) Assessment of anticoagulation in patients receiving ultrasound-assisted catheter-directed thrombolysis for treatment of pulmonary embolism. Ann Pharmacother 53(5):453–457. 10.1177/1060028018811155 30378437 10.1177/1060028018811155

[5] Kamal AH, Tefferi A, Pruthi RK (2007) How to interpret and pursue an abnormal prothrombin time, activated partial thromboplastin time, and bleeding time in adults. Mayo Clin Proc 82(7):864–873. 10.4065/82.7.864 17605969 10.4065/82.7.864

[6] Prucnal CK, Jansson PS, Deadmon E, Rosovsky RP, Zheng H, Kabrhel C (2020) Analysis of partial thromboplastin times in patients with pulmonary embolism during the first 48 hours of anticoagulation with unfractionated heparin. Acad Emerg Med 27(2):117–127. 10.1111/acem.13872 31625654 10.1111/acem.13872

[7] Wheeler AP, Jaquiss RD, Newman JH (1988) Physician practices in the treatment of pulmonary embolism and deep venous thrombosis. Arch Intern Med 148(6):1321–1325 3377615

[8] Bach AG, Taute BM, Baasai N et al (2016) 30-day mortality in acute pulmonary embolism: prognostic value of clinical scores and anamnestic features. PLoS ONE 11(2):e0148728. 10.1371/journal.pone.0148728 26866472 10.1371/journal.pone.0148728 PMC4750907

[9] Smith SB, Geske JB, Maguire JM, Zane NA, Carter RE, Morgenthaler TI (2010) Early anticoagulation is associated with reduced mortality for acute pulmonary embolism. Chest 137(6):1382–1390. 10.1378/chest.09-0959 20081101 10.1378/chest.09-0959 PMC3021363

[10] Ting C, Sylvester KW, Schurr JW (2018) Time in the therapeutic range for assessing anticoagulation quality in patients receiving continuous unfractionated heparin. Clin Appl Thromb Hemost 24(9_suppl):178S-181S. 10.1177/1076029618798944 30213200 10.1177/1076029618798944 PMC6714859

[11] Campbell NR, Hull RD, Brant R, Hogan DB, Pineo GF, Raskob GE (1998) Different effects of heparin in males and females. Clin Invest Med 21(2):71–78 9562927

[12] Roosendaal LC, Wiersema AM, Smit JW, Doganer O, Blankensteijn JD, Jongkind V (2022) Editor’s choice—sex differences in response to administration of heparin during non-cardiac arterial procedures. Eur J Vasc Endovasc Surg 64(5):557–565. 10.1016/j.ejvs.2022.08.005 35973666 10.1016/j.ejvs.2022.08.005

[13] Streng AS, Delnoij TSR, Mulder MMG et al (2020) Monitoring of unfractionated heparin in severe COVID-19: an observational study of patients on CRRT and ECMO. TH Open 4(4):e365–e375. 10.1055/s-0040-1719083 33235946 10.1055/s-0040-1719083 PMC7676995

[14] Zehnder J, Price E, Jin J (2012) Controversies in heparin monitoring. Am J Hematol 87(Suppl 1):S137–S140. 10.1002/ajh.23210 22495972 10.1002/ajh.23210

[15] Bĕlohlávek J, Dytrych V, Linhart A (2013) Pulmonary embolism, part I: epidemiology, risk factors and risk stratification, pathophysiology, clinical presentation, diagnosis and nonthrombotic pulmonary embolism. Exp Clin Cardiol 18(2):129–138 23940438 PMC3718593

[16] Eikelboom JW, Hirsh J (2006) Monitoring unfractionated heparin with the aPTT: time for a fresh look. Thromb Haemost 96(5):547–552 17080209

[17] Raschke R, Hirsh J, Guidry JR (2003) Suboptimal monitoring and dosing of unfractionated heparin in comparative studies with low-molecular-weight heparin. Ann Intern Med 138(9):720–723. 10.7326/0003-4819-138-9-200305060-00008 12729426 10.7326/0003-4819-138-9-200305060-00008

[18] Levy JH, Connors JM (2021) Heparin resistance—clinical perspectives and management strategies. N Engl J Med 385(9):826–832. 10.1056/NEJMra2104091 34437785 10.1056/NEJMra2104091

[19] Toulon P, Smahi M, De Pooter N (2021) APTT therapeutic range for monitoring unfractionated heparin therapy: significant impact of the anti-Xa reagent used for correlation. J Thromb Haemost 19(8):2002–2006. 10.1111/jth.15264 33555096 10.1111/jth.15264

[20] Swayngim R, Preslaski C, Burlew CC, Beyer J (2021) Comparison of clinical outcomes using activated partial thromboplastin time versus antifactor-Xa for monitoring therapeutic unfractionated heparin: a systematic review and meta-analysis. Thromb Res 208:18–25. 10.1016/j.thromres.2021.10.010 34678527 10.1016/j.thromres.2021.10.010

