# Supplementary material for: Impact of time in therapeutic range (TTR) within the first 72 h on prognosis in patients with pulmonary embolism treated with unfractionated heparin
Source: J Thromb Thrombolysis. 2025 Aug 14;59(1):188–95. doi: 10.1007/s11239-025-03167-2 (PMC12886203; doi:10.1007/s11239-025-03167-2)
Supplement: Supplementary file 1 — Supplementary file1 (DOCX 17 kb) [file 11239_2025_3167_MOESM1_ESM.docx]

**Table S1** Heparin administration protocol

| **PTT**  **(Seconds)** | **Bolus**  **(Units)** | **Stop administration**  **(Minutes)** | **Change of administration rate**  **(Units/Hour)** | **Change of administration rate**  **(Ml/Hour)** | **Next PTT Test (Hours)** |
| --- | --- | --- | --- | --- | --- |
| 35> | 3000 | 0 | + 200 | + 0.4 | 4-6 |
| 35-59 | 1500 | 0 | + 150 | + 0.3 | 4-6 |
| 60-85 | 0 | 0 | No Change | No Change | 4-6 |
| 86-100 | 0 | 0 | - 100 | - 0.2 | 4-6 |
| 101-120 | 0 | 0 | - 150 | - 0.3 | 4-6 |
| 121-150 | 0 | 0 | - 200 | - 0.4 | 4-6 |
| 150< | 0 | 60 | According to evaluation |  | 4-6 |

- Initial dose in pulmonary embolism – 18 units/kg/hour (see protocol).
- 80 units/kg should be given as a bolus and then continuous administration should be initiated.
- PTT levels should be checked every 4-6 hours until target dose is achieved and then checked at least once daily.
